# Preparation of PANI/CuPc/PDMS Composite Elastomer with High Dielectric Constant and Low Modulus Assisted by Electric Fields

**DOI:** 10.3390/polym16111549

**Published:** 2024-05-30

**Authors:** Jinjin Hu, Beizhi Chu, Xueqing Liu, Huaixiao Wei, Jianwen Wang, Xue Kan, Yumin Xia, Shuohan Huang, Yuwei Chen

**Affiliations:** 1 Key Laboratory of Rubber-Plastics, Ministry of Education/Shandong Provincial Key Laboratory of Rubber-Plastics, Qingdao University of Science & Technology, Qingdao 266042, China; 15137596272@163.com (J.H.); weihuaixiao2022@163.com (H.W.); 15266728568@163.com (J.W.); 15269640882@163.com (X.K.); 2 Key Laboratory of Optoelectronic Chemical Materials and Devices, Ministry of Education and Flexible Display Materials and Technology Co-Innovation Centre of Hubei Province, Jianghan University, Wuhan 430056, China; liuxueqing2000@163.com; 3 State Key Laboratory for Modification of Chemical Fibers and Polymer Materials, College of Materials Science and Engineering, Donghua University, Shanghai 201600, China; xym@dhu.edu.cn (Y.X.); huangs@dhu.edu.cn (S.H.)

**Keywords:** electric fields assembly, silicone rubber, dielectric constant, elastic modulus, electro-deformation

## Abstract

Dielectric elastomer is a kind of electronic electroactive polymer, which plays an important role in the application of soft robots and flexible electronics. In this study, an all-organic polyaniline/copper phthalocyanine/silicone rubber (PANI/CuPc/PDMS) dielectric composite with superior comprehensive properties was prepared by manipulating the arrangement of filler in a polymer matrix assisted by electric fields. Both CuPc particles and PANI particles can form network structures in the PDMS matrix by self-assembly under electric fields, which can enhance the dielectric properties of the composites at low filler content. The dielectric constant of the assembled PANI/CuPc/PDMS composites can reach up to 140 at 100 Hz when the content of CuPc and PANI particles is 4 wt% and 2.5 wt%, respectively. Moreover, the elastic modulus of the composites remains below 2 MPa, which is important for electro-deforming. The strain of assembled PANI/CuPc/PDMS three-phase composites at low electric field strength (2 kV/mm) can increase up to five times the composites with randomly dispersed particles, which makes this composite have potential application in the field of soft robots and flexible electronics.

## 1. Introduction

Electroactive polymers (EAPs) can be divided into ionic EAPs and electronic EAPs according to their driving mechanisms [1,2,3]. Ionic EAPs respond to the electric field by the diffusion of ions in the material, which requires an electrolyte environment to work, and hence, limits their applications in dry or extreme environments [4,5]. Compared with ionic EAPs, electronic EAPs have the advantages of a wide linear response range, high flexibility, long life, good reliability, and high efficiency [6,7]. Dielectric elastomers (DEs) are a kind of electronic EAPs, which have the characteristics of high elastic energy density, ultra-short response time, and flexibility, and can convert electrical energy into mechanical energy under electric fields, which have been widely used in the field of soft robots, wearable electronics, and human/machine interface equipment [8,9,10,11].

However, there are challenges that demand prompt solutions. Existing DEs usually have an inherent low dielectric constant (ε = 2–10), which makes it difficult to meet the high driving voltage (>100 kV·mm^−1^). In order to generate a larger drive at a relatively low driving voltage, the dielectric constant can be increased by adding a high dielectric filler, such as ceramic fillers (BaTiO_3_, SnO_2_, and Al_2_O_3_) or conductive fillers (carbon fibers, carbon black, metal powder, polyaniline, and polypyrrole) and so on [12,13,14,15,16,17]. The composites filled with ceramic particles require higher filler content to achieve a high dielectric constant, and this results in decreased flexibility [18]. For the composites filled with conductive fillers, the dielectric loss increases rapidly as the dielectric constant increases since the percolation threshold exists, which brings instability to practical applications. Therefore, the composites filled with two or more types of fillers have been explored [19,20]. By introducing Ag-coated ceramic filler K_2_Ni_0.93_Ti_7.07_O_16_ (KNTO) into polyvinylidene fluoride (PVDF), the dielectric constant of 2.5 wt% KNTO @ Ag/PVDF composites was 1.62 times that of KNTO-PVDF and 2.59 times of pure PVDF film [21]. Huanhuan Zhang [22] reported that the addition of two-dimensional organic modified montmorillonite (OMMT) into CNT/PVDF composites can prevent the formation of CNT conductive pathways and allow electrons to move freely in CNT clusters within a short distance. This is because conductive CNTs can form a multitude of microcapacitors within the insulating medium of PVDF. The polarization between CNTs and PVDF leads to the accumulation of charge, which ultimately results in an increase in the dielectric constant.

In order to further improve the dielectric constant of composites, researchers have started to explore external fields, such as the shear field, electric field, and magnetic field, to control the distribution of fillers in the polymer matrix [23,24,25]. Micro charge storage structures can be formed by orientation so that the dielectric properties are improved. Ting Chen [26] assembled sodium carboxymethyl cellulose (CMC) microfibers in a biocompatible silicone elastomer PDMS under a DC electric field. The prepared composite film with CMC (15 wt%) arrangement showed a significant dielectric constant, almost seven times higher than its counterparts. Weifei Wu [19] also proposed the strategy of using micro-sized boron nitride (BN) and nano-sized alumina (Al_2_O_3_) to enhance the dielectric properties of PDMS.

PDMS is the most commonly used dielectric elastomer due to its high reactivity, low Young’s modulus, low price, and excellent plasticity [27,28]. The fluidity of the PDMS prepolymer can be well applied to the external field to regulate the particle arrangement process, and the rapid curing after heating can well maintain the orientation structure of the particles [29,30]. PDMS is a non-polar rubber [31]. Due to its low energy density [32,33], the driving strain of PDMS is usually very small. By adding high dielectric fillers and designing the internal structure of the material to prepare a composite dielectric elastomer, the driving strain can be improved.

In this study, organic conductive PANI and semiconductor CuPc particles were chosen as fillers to enhance the dielectric properties of PDMS by electric field manipulation. The effect of electric strength and frequency on the self-assembly process of both PANI and CuPc are investigated by optical microscopy. The increase in electric field intensity and time are beneficial to the establishment of the network structure of PANI particles in the PDMS matrix, and the greater the electric field intensity, the shorter the time required to form a network structure. The synergistic effect of PANI and CuPc particles can improve the dielectric constant of the composite films effectively. The dielectric constant of the assembled PANI/CuPc/PDMS three-phase composite film can reach 140 at 100 Hz when the content of CuPc is 4 wt% and the content of PANI is 2.5 wt%. The modulus of the three-phase composite film of PANI/CuPc/PDMS is kept below 2 MPa. The high dielectric constant and low modulus properties make this composite a good candidate for electro-deformation materials. The strain at low electric field strength (2 kV/mm) of the self-assembled PANI/CuPc/PDMS three-phase composite films increased by nearly five times.

## 2. Materials and Methods

### 2.1. Materials

Polydimethylsiloxane (SILASTICTM, rbr-9101, 1.05 g/cm^3^) and curing agent were purchased from Dow Corning in the Midland, TX, USA. Polyaniline powder was supplied by Guangzhou Haoxuan Chemical Co., Ltd., in Guangzhou, China. Copper phthalocyanine powder was purchased from Anhui Kuer Biological Engineering Co., Ltd., in Hefei, China. Anhydrous ethanol (analytically pure) was obtained from Shanghai McLean Biochemical Technology Co., Ltd., in Shanghai, China. Conductive silver paste (0.3 mL) was brought from Shenzhen Wavis Electronic Technology Co., Ltd., in Shenzhen, China.

### 2.2. Preparation of PANI/PDMS and CuPc/PDMS Two-Phase Composite Films

A total of 10 g PDMS and 1 g curing agent were put into a disposable plastic cup, and then PANI particles and CuPc particles with different mass fraction contents were weighed and put into the mixed matrix. The mixture was evenly mixed by a non-intrusive centrifuge. Firstly, it was rotated at 500 r/min for 2 min in non-vacuum mode, and then it was rotated at 1200 r/min for 2 min in vacuum mode. The mixed and defoamed suspension was poured into a square mold with a thickness of 1 mm composed of two ITO-coated conductive glass electrodes. The top electrode was connected to a high-voltage amplifier, the bottom was grounded, and the electric field voltage size, frequency, and time were set. The PANI/PDMS and CuPc/PDMS composite membranes were prepared by thermal curing at 100 °C for one hour on a heating stage, and then the membranes were removed by cooling. In the absence of an electric field, the PANI/PDMS and CuPc/PDMS composite membranes were prepared by the same experimental method.

### 2.3. Preparation of PANI/CuPc/PDMS Three-Phase Composites

**Figure 1:**
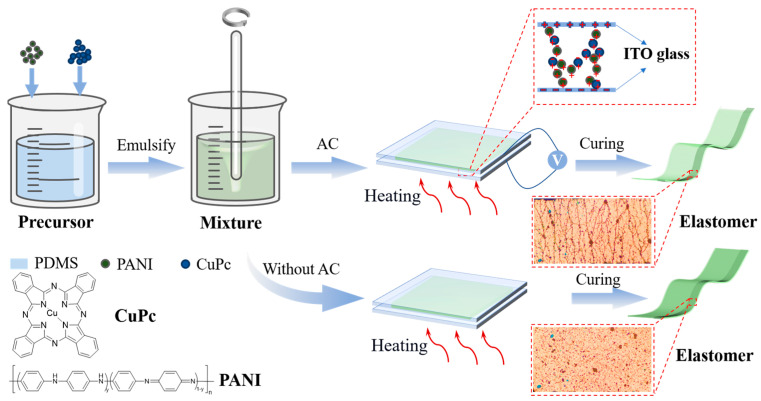
Preparation of PANI/CuPc/PDMS three-phase composite membrane.

The weight ratio of the PDMS prepolymer to the curing agent was 10:1, and then the PANI particles with a mass fraction of 2.5 wt% and CuPc particles with different mass fractions (from 1 wt% to 5 wt%) were added to the matrix. The two filler particles were added to the PDMS matrix, and the subsequent experimental steps were the same as the preparation process of the two-phase composite membrane. In the absence of an electric field, the PANI/CuPc/PDMS composite membrane was prepared by the same experimental method. The preparation process is shown in Figure 1.

### 2.4. Characterization Methods

The MP41 optical microscope from Guangdong Mingmei Technology Co., Ltd. (Guangzhou, China) was used to observe the movement process of different particles in the PDMS matrix under the action of an AC electric field. The cross-sectional morphology of the composite film was observed by using Phenom’s ProX scanning electron microscope (SEM) which is manufactured by Japanese electronics, Tokyo, Japan. Before the test, the cross-section of the sample needs to be sprayed with gold, and the test acceleration voltage is 10 kV. Finally, a picture of the internal structure of the composite film can be obtained. The mechanical properties of the composite films were tested by Zwick z005 tensile testing machine. The composite film was cut into a dumbbell-shaped sample of 115 mm × 6 mm using a standard cutter. The upward tensile rate of the universal tensile machine was set to 20 mm·min^−1^ during the experiment. Each spline was tested five times and the intermediate value was taken. Novocontrol’s Alpha-A dielectric impedance spectrometer was used to test the dielectric constant. Before the test, the sample was cut into a circular slice with a circular cutter with an inner diameter of 25 mm, and a circular copper slice with a diameter of 30 mm was used as the electrode. We applied a frequency-varying alternating current (AC) signal to the electrodes and used a data acquisition device to record the data. In the fabrication of dielectric elastomer actuators, the composite film was cut into a disc with a diameter of 25 mm and a thickness of 1 mm. It was fixed at the edges with a self-made PMMA clamp. Conductive silver paste with a diameter of 5 mm was evenly applied to both surfaces of the composite film. The film, now coated with double-sided electrodes, was placed in a laser interferometer that was coupled with a ferroelectric analyzer. Voltage was applied without pre-stress until the sample underwent electrical breakdown.

## 3. Results

### 3.1. Orientation Kinetics of PANI Particles and CuPc Particles under Electric Fields

The self-assembly process of particles under electric fields is mainly affected by the intensity of polarization and the interaction between particles, which are induced by electric field parameters. In order to explore the optimal self-assembly parameters, an optical microscope was used to observe the effect of electric field intensity, frequency, and time on the orientation structure in situ.

#### 3.1.1. Effect of Electric Field Intensity and Time on the Self-Assembly Process of PANI Particles

**Figure 2:**
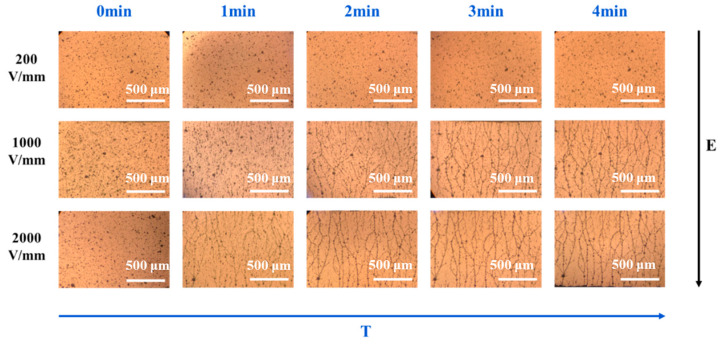
In situ optical microscope images of the self-assembly process of the PANI particles in PDMS under electric fields with different electric field intensities and times. (The scale in the figure is 500 μm).

As shown in Figure 2, when the electric field strength is 200 V/mm, the particles are uniformly dispersed in the matrix and no micro-structures are formed during the 4 min period of applying electric fields. However, when the electric field intensity increases from 200 V/mm to 1000 V/mm, the orientation structure of the PANI particles gradually forms. A large number of the dispersed PANI particles still exist in the matrix, and the formed micro-network structures are relatively sparse. When the electric field strength increases to 2000 V/mm, the network structure of the particles in the matrix gradually becomes perfect and compact with the increase in time. Therefore, it can be concluded that the increase in electric field intensity and time are beneficial to the establishment of the network structure of the PANI particles in the PDMS matrix, and the greater the electric field intensity, the shorter the time required to form a network structure.

#### 3.1.2. Effect of Electric Field Frequency on the Self-Assembly Process of PANI Particles

**Figure 3:**
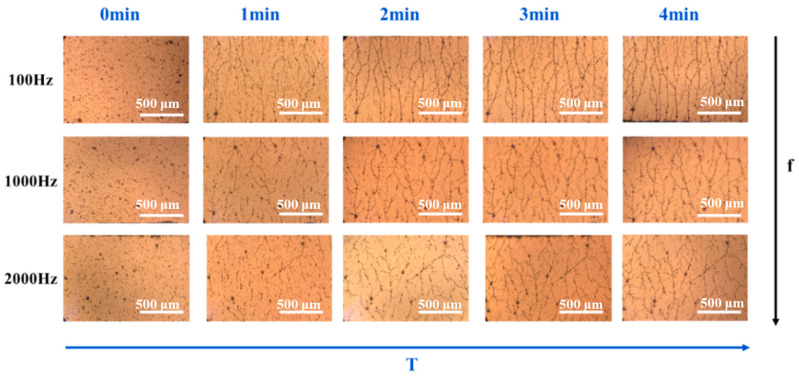
In situ optical microscope images of the PANI particles in the PDMS matrix under electric fields with different frequencies. (The scale in the figures is 500 μm).

The effect of frequency on the formation of polyaniline PANI network structure was investigated under an electric field of 2000 V/mm. As shown in Figure 3, when the electric field frequency is 100 Hz and the orientation time is 1 min, the PANI particles slowly begin to form a short chain structure from both sides of the electrodes. At 2 min, the short chain structures are gradually connected to form chain structures. With the extension of time, the surrounding particles are continuously connected to the long chains and dendritic structures are formed. Finally, the short chain structure overlaps with each other and the long chain structure forms a more compact network structure in the matrix. According to Figure 3, when the electric field frequency is 2000 Hz, the network and structure formed by the PANI particles are relatively loose, because the PANI particles do not have time to respond to the changes in the electric field, resulting in a decrease in orientation. At an electric field frequency of 100 Hz, the network structure formed by the PANI particles is denser and the self-assembly structure is more perfect. This is because PANI has enough time to respond to the changes in the electric field at 100 Hz, which is conducive to the formation of a dense structure.

#### 3.1.3. Characterization of the Effect of Electric Field on CuPc Particles

**Figure 4:**
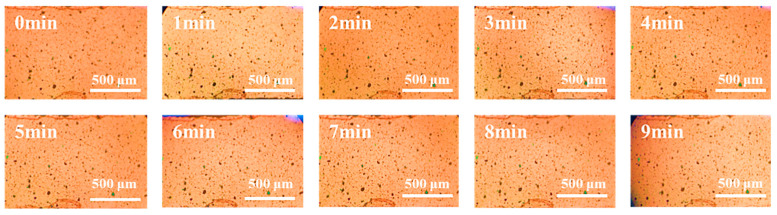
In situ images of the CuPc particles in the PDMS matrix by optical microscopy under electric fields. (The scale in the figure is 500 μm).

The orientation of the CuPc particles was also observed under the electric field (100 Hz, 2000 V/mm). As shown in Figure 4, The CuPc particles could be slightly polarized and some short chain structures were formed, but there was no large-scale movement of the particles. After 9 min treatment under electric fields, there were still no network structures like the PANI particles formed, which is mainly because CuPc has a high π-π conjugated structure, which makes the electron cloud distribution relatively uniform.

#### 3.1.4. Orientation Process of PANI Particles and CuPc Particles under Electric Field

**Figure 5:**
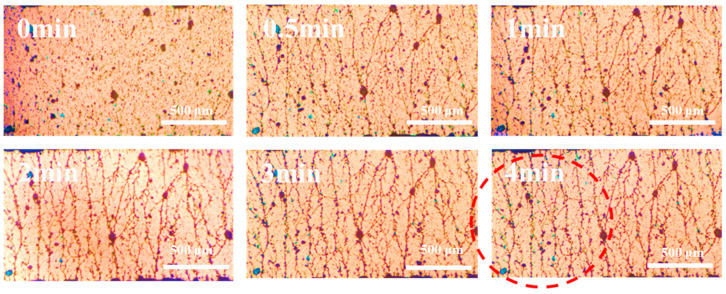
In situ images of the PANI and CuPc particles in the PDMS matrix under electric fields. (The scale in the figure is 500 μm).

Through the exploration of electric field parameters, it was found that when the electric field intensity was 2000 V/mm and the electric field frequency was 100 Hz, the self-assembly of the particles was more efficient than under other parameters, and the formed network structure was relatively perfect and close. The electric field under this condition was used to orient the PANI and CuPc particles in the matrix at the same time. It can be seen from Figure 5, at the beginning, the particles overlap with each other to form a short chain structure. Then, the short chain structures are connected to each other to form a long chain structure in 30 s. After 1 min, the short chain structures intersect with each other and network structures are formed, and this chain structure becomes more perfect as time goes on, which is highlighted in the red circle.

### 3.2. Characterization of PANI/PDMS and CuPc/PDMS Composite Films

In order to understand the synergistic effect between the PANI particles and CuPc particles and to analyze the effect of the self-assembled micro-structures on the performance of the composite films, the properties of the two-phase PANI/PDMS composite film and CuPc/PDMS composite film were studied first.

#### 3.2.1. Analysis of Cross-Section Morphology of PANI/PDMS Composite Film

**Figure 6:**
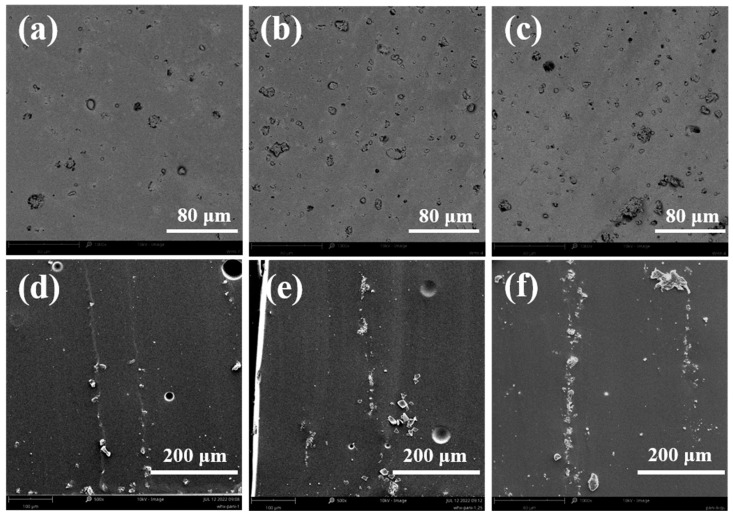
Scanning electron microscopy of PANI/PDMS composite films: (**a**) 1% PANI/PDMS, (**b**) 2.5% PANI/PDMS, and (**c**) 5% PANI/PDMS composite films prepared without electric field; (**d**) 1% PANI/PDMS, (**e**) 2.5% PANI/PDMS, and (**f**) 5%PANI/PDMS composite films prepared under electric field.

Figure 6 shows the scanning electron microscope images of the fracture surface of the PANI/PDMS composite films prepared with and without electric fields. Figure 6a–c show the PANI/PDMS composite film prepared without electric fields. It can be seen that the PANI particles in PDMS are uniformly distributed. Compared with the size of the PANI particles themselves, the distance between the particles in the matrix is larger. Figure 6d–f show the scanning electron microscope image of the cross-section of the PANI/PDMS composite film prepared under electric fields, the distance between the PANI particles in the PDMS matrix is shortened and chain-like structures are formed. With the increase in the PANI particle content, more oriented structures can be observed and the formed oriented structures are more robust.

#### 3.2.2. Analysis of Dielectric Properties of PANI/PDMS Composite Films

**Figure 7:**
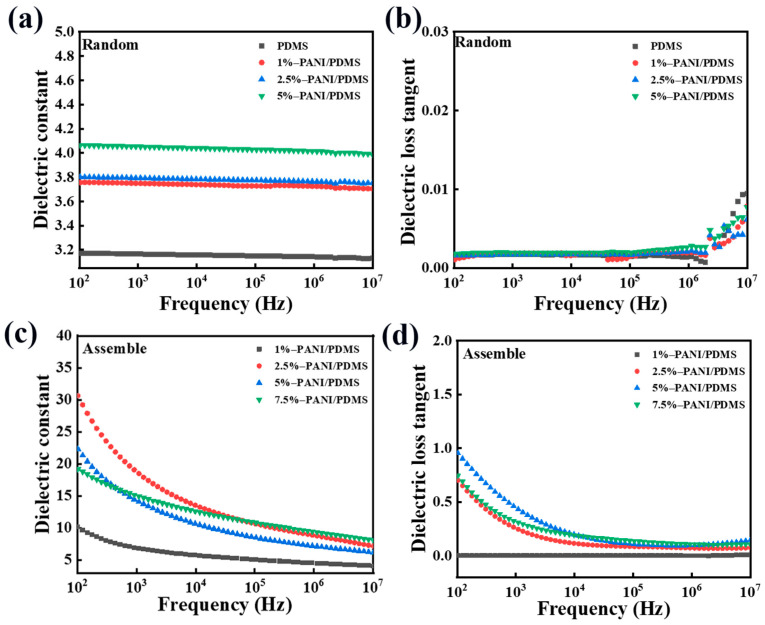
Dielectric properties of the unoriented PANI/PDMS composite films with different PANI contents: (**a**) the variation in dielectric constant with frequency and (**b**) the variation in dielectric loss with frequency. The dielectric properties of the PANI/PDMS composite films with different PANI contents in orientation: (**c**) the dielectric constant changes with frequency and (**d**) the dielectric loss changes with frequency.

The dielectric constant of the PANI/PDMS composite film prepared with and without electric fields increases with the decrease in frequency, but the upward trend of the composite film prepared under an electric field is more obvious. In Figure 7a, the dielectric constant of the 5 wt% PANI/PDMS composite film prepared without an electric field can reach higher than 4.0 at the test frequency of 100 Hz, while the dielectric constant of pure PDMS is below 3.2. The dielectric constant of all PANI/PDMS composite films increases with the increase in the proportion of the particles in the matrix, mainly because PANI is equivalent to multiple electrodes in the matrix. When the filler content increases, the capacitor effect increases, so the dielectric constant increases. Because the PANI in the matrix is too far away from each other and is separated by the insulating PDMS, the dielectric constant is increased but the increase is not large. With the same content of PANI, there are network structures inside the PANI/PDMS composite film prepared under the electric field, and the dielectric constant increases more obviously. As shown in Figure 7c, the dielectric constant of the 2.5 wt% PANI/PDMS composite film prepared under the electric field condition is 30 at 100 Hz, which is 7.5 times higher than that of the randomly dispersed 2.5 wt% PANI/PDMS composite film and nearly 10 times higher than that of pure PDMS. Obviously, this method of constructing an internal network structure is very effective for the increase in dielectric constant. The increase in dielectric constant is mainly because polyaniline forms a large number of microcapacitors and short conductive chains in the matrix, which can improve the overall dielectric constant of the composite film.

From the comparison of Figure 7b,d, it is found that the dielectric loss of the composite film prepared under the electric field is higher than that without the electric field, and increases with the decrease in the frequency. Although the network structure inside the film increases the dielectric loss, the dielectric loss of the PANI/PDMS composite film is still low. As shown in Figure 7d, the dielectric loss of the 5 wt% PANI/PDMS composite film prepared under the electric field is about 0.85 at the test frequency of 100 Hz, which is a relatively low value.

**Figure 8:**
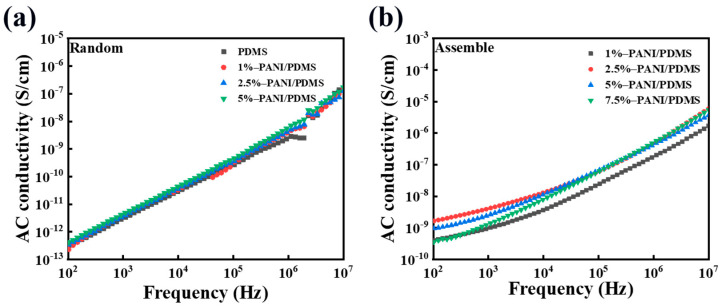
(**a**) The variation in electrical conductivity with frequency for the PANI/PDMS composite films with different PANI contents that are not oriented and (**b**) the curve of electrical conductivity with frequency for the PANI/PDMS composite films with different PANI contents that are oriented.

In order to explore the insulation properties of the composite film, the conductivity of the material was tested. In the whole frequency range, the conductivity increases with the increase in frequency. This is because as the frequency increases, the number of polarization within a certain period of time increases. This leads to increased charge movement within the composite material, increasing its conductivity. As shown in Figure 8a, the conductivity of the PANI/PDMS composite film prepared without an electric field is basically unchanged, which is almost the same as that of pure PDMS. In Figure 8b, the conductivity of the PANI/PDMS composite film prepared by applying an electric field is higher than that of the PANI/PDMS composite film prepared without an electric field. At the testing frequency of 10^2^–10^7^ Hz, the conductivity of the composite film is mostly maintained below 10^−6^ S/cm, which is within the conductivity range of the insulator. The prepared composite film has excellent insulation ability.

#### 3.2.3. Characterization of Cross-Section Morphology of CuPc/PDMS Composite Films

**Figure 9:**
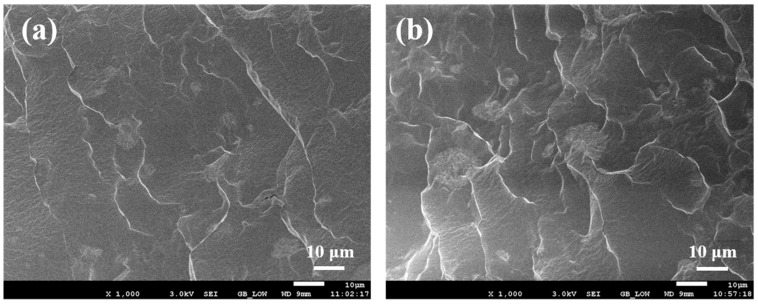
Scanning electron microscopy of CuPc/PDMS composite films: (**a**) 2.5% CuPc/PDMS and (**b**) 5% CuPc/PDMS.

As shown in Figure 9, the CuPc particles are well dispersed in the PDMS matrix in the SEM images of the CuPc/PDMS composite films with different contents of the CuPc particles. With the increase in the content of the CuPc particles, the distance between the particles is reduced, but the distance between the particles is still large relative to the size of the particles themselves.

#### 3.2.4. Analysis of Dielectric Properties of CuPc/PDMS Composite Films

**Figure 10:**
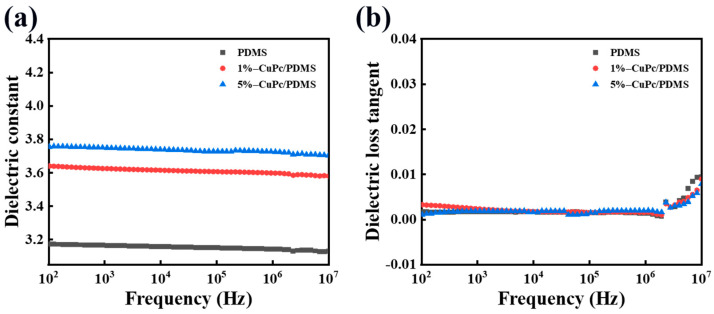
Dielectric properties of CuPc/PDMS composite films with different CuPc contents: (**a**) dielectric constant change with frequency and (**b**) dielectric loss curve change with frequency.

Figure 10 shows the dielectric properties of the CuPc/PDMS composite films with different CuPc content. The dielectric constant of the composite film changes with the frequency. With the increase in the CuPc particle content, the dielectric constant of the composite film increases gradually. When the content of the CuPc particles is 5 wt%, the dielectric constant increases from 3.2 to 3.8. Because many charge flows in the molecule are hindered by the insulating layer components, charge delocalization is formed. This is equivalent to the capacitor effect of the internal boundary layer formed by the core and insulating layer of the semiconductor. The electrons containing conjugated π bonds in the whole molecule are very easy to shift in the electric field, so they have a high dielectric response. With the addition of the CuPc particles, the dielectric loss of the composite material increases slightly, but the overall increase is not large. Because the particle mass fraction is small, the distance between the particles in the matrix is large, and the insulating PDMS also blocks the direct contact between the particles.

### 3.3. PANI/CuPc/PDMS Three-Phase Composite Film

Based on the previous research on the dielectric properties of the PANI/PDMS composite film and CuPc/PDMS composite film, the experimental research and analysis of the PANI/CuPc/PDMS three-phase composite film were continued.

#### 3.3.1. Characterization of the Cross-Section Morphology of PANI/CuPc/PDMS Composite Film

**Figure 11:**
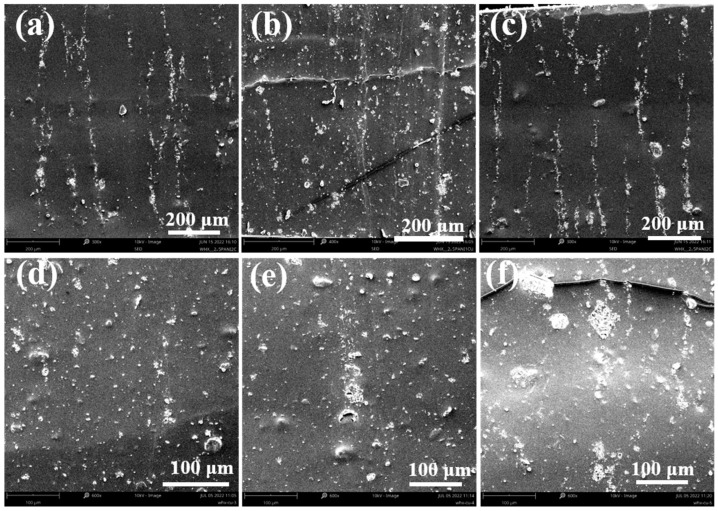
Scanning electron microscopy of PANI/CuPc/PDMS composites: (**a**) 2.5% PANI/PDMS, (**b**) 2.5% PANI/1% CuPc/PDMS, (**c**) 2.5% PANI/2% CuPc/PDMS, (**d**) 2.5% PANI/3% CuPc/PDMS, (**e**) 2.5% PANI/4% CuPc/PDMS, and (**f**) 2.5% PANI/5% CuPc/PDMS.

Figure 11a is the cross-section scan of the 2.5% PANI/PDMS. Compared with the PANI/CuPc/PDMS composite film with the CuPc particles, the formed chain segment structure is thinner and the distance between the chain segments is relatively far. The chain structure formed by the composite film containing CuPc is relatively thick and the distance between the particles is relatively close. Among them, the 2.5%PANI/4%CuPc/PDMS composite film in Figure 11e forms the most complete orientation structure in the matrix.

#### 3.3.2. Analysis of Dielectric Properties and Electrical Conductivity of PANI/CuPc/PDMS Composite Films

**Figure 12:**
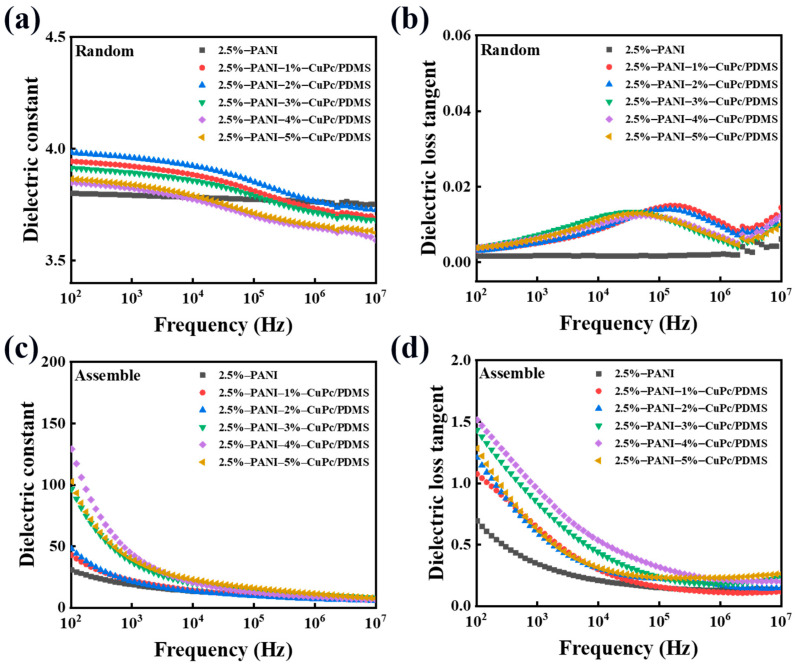
Dielectric properties of the PANI/CuPc/PDMS composite films. The dielectric constant (**a**) and the dielectric loss (**b**) of random PANI/CuPc/PDMS composite films, and the dielectric constant (**c**) and dielectric loss (**d**) of the assembled PANI/CuPc/PDMS composites.

Figure 12a,b show the trend of the dielectric constant and dielectric loss of the PANI/CuPc/PDMS composite film prepared without an electric field changing with frequency. Due to the addition of the CuPc particles in the PANI/PDMS composite film, the dielectric constant of the composite film increases with the increase in the filler content. When the content of CuPc is 5 wt%, the maximum dielectric constant of the PANI/CuPc/PDMS three-phase composite film is about 4, which is greater than that of the PANI/PDMS composite film. The curve in Figure 12a fluctuates slightly in the wide frequency range of 10^2^ Hz to 10^7^ Hz. This may be because the particles have a good combination with the matrix at a low filler loading, and the filler in the polymer matrix limits the mobility of the molecular chain and limits the movement of the polymer molecular chain. Figure 12b shows the curve of the dielectric loss versus frequency of the PANI/CuPc/PDMS three-phase composite film without electric field orientation. All the composite films were maintained at a low level in a wide frequency range from 10^2^ Hz to 10^7^ Hz, and there was a small wave peak from 10^4^ Hz to 10^6^ Hz, which was caused by the emergence of slow polarization. Figure 12c,d show the relationship between the dielectric constant and dielectric loss of the oriented PANI/CuPc/PDMS composite film prepared under the applied electric field and the frequency change. The dielectric constant of the composite film increases significantly with the increase in the CuPc filler particle content. When the content of the CuPc filler increased to 4 wt%, the dielectric constant of the oriented PANI/CuPc/PDMS composite film increased to 140 at 100 Hz. The dielectric loss of the oriented PANI/CuPc/PDMS composite film is higher than that of the two-phase composite film without the CuPc particles, but the overall increase range is relatively small. The dielectric constant of the composite film increases with the decrease in frequency. On the one hand, PANI is oriented to a network structure composed of single particles under the action of an electric field, and the interfacial polarization increases. On the other hand, the electron displacement of the whole molecule of the conjugated π bond of the CuPc particle becomes larger in the electric field, and the two particles cooperate with each other, so the dielectric constant increases with the decrease in frequency. The reason why the dielectric loss decreases with the increase in frequency is that the frequency changes rapidly, and the movement ability of the charge in the molecule and the interface charge cannot keep up, resulting in a decrease in dielectric loss.

**Figure 13:**
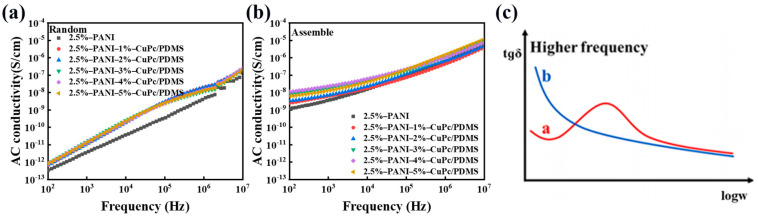
(**a**) Conductivity of the non-assembled PANI/CuPc/PDMS composite films, (**b**) the conductivity of the assembled PANI/CuPc/PDMS composite films, and (**c**) the relationship between dielectric loss tgδ and the logarithm of angular frequency logw at higher frequencies. In the low-frequency range, the relationship between tgδ and *w* in the tape is like line b, and when the frequency is higher, the relationship between tgδ and *w* is like line a.

Figure 13a,b show the frequency dependence of the conductivity of the unoriented PANI/CuPc/PDMS composite film and the assembled PANI/CuPc/PDMS composite film. The conductivity of the PANI/CuPc/PDMS composite film after electric field assembly is greatly improved compared with the non-assembled composite films, and the conductivity is increased from 10^−12^ S/cm to 10^−8^ S/cm at 100 Hz. In Figure 13b, the conductivity of all composite films is between 10^−8^ S/cm and 10^−5^ S/cm. The dielectric loss of the composite film is also related to the conductivity of the film. Based on the Debye equation and the Kramers/Kröning relationship, the relationship between the dielectric loss and the angular frequency w can be obtained. Equation (1) is as follows:

(1) $$tg\delta =\frac{\frac{\gamma }{\varepsilon_{0}}+w\left(\varepsilon_{rs}-\varepsilon_{r\infty }\right)\frac{w\tau }{1+w^{2}\tau^{2}}}{w\left[\varepsilon_{rs}+\frac{\varepsilon_{rs}-\varepsilon_{r\infty }}{1+w^{2}\tau^{2}}\right]}$$

In the formula, tgδ is the dielectric loss, w is the angular frequency, γ is the conductivity of the film, τ is the relaxation time constant, $\xi_{rs}$ is the relative dielectric constant when the angular frequency *w* approaches 0, and $\xi_{r\infty }$ is the relative dielectric constant when the angular frequency *w* approaches ∞.

When the frequency of the external environment is relatively low, the term of $w^{2}\tau^{2}$ or wτ can be approximately omitted in the equation, so the dielectric loss at this time is basically caused by the leakage current. Equation (2) can be changed into the following:

(2) $$tg\delta \approx \frac{\gamma }{w\xi_{0}\xi_{rs}}$$

From the above formula, it can be concluded that in the lower frequency range, tgδ decreases with the increase in frequency. When the frequency is relatively high, the dielectric loss of the composite film is shown in the curve of a line in Figure 13c. There is also another phenomenon that when the frequency continues to increase, the dielectric loss of the material in some specific frequency ranges may continue to rise to a peak due to some slow polarization. This phenomenon is called anomalous dispersion. However, if the conductivity of the composite film is large enough, the peak value of the dielectric loss may be completely covered. Compared with the conductivity of (a) and (b) in Figure 13, the conductivity of the oriented composite film is larger, which is also the reason why the dielectric loss curves of (b) and (d) in Figure 13 are different.

#### 3.3.3. Mechanical Property Analysis of PANI/CuPc/PDMS Composite Film

**Figure 14:**
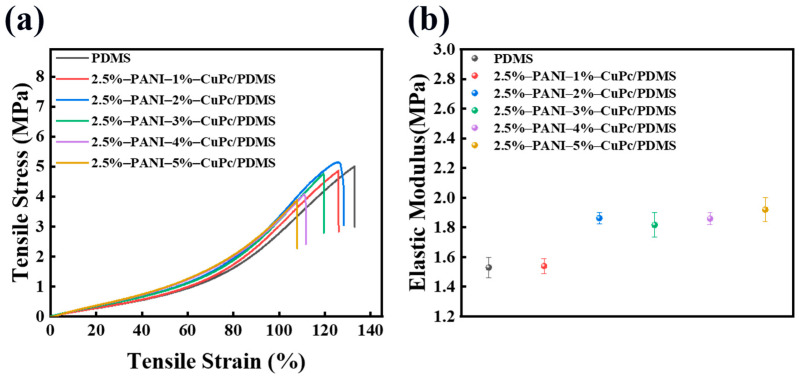
Tensile properties of PANI/CuPc/PDMS composite films: (**a**) stress/strain diagram and (**b**) elastic modulus diagram.

It can be observed from Figure 14a that the elongation at the break of the composite film with filler particles is lower than that of pure PDMS. This is because the addition of the PANI particles and CuPc particles hinders the movement of the molecular chains in PDMS, and the particles have a certain interaction with the PDMS matrix, so the elongation at the break of the composite film decreases. Figure 14b shows the change in the elastic modulus of the composite film after adding the particles. The elastic modulus of the composite film increases with the addition of the filler particles. This is because the filler particles after the electric field orientation form a network structure in the matrix, which has a great influence on the elastic modulus. However, due to the small particle content, the elastic modulus of all composite films remains below 2 MPa.

#### 3.3.4. Analysis of Electro-Deformation Properties of PANI/CuPc/PDMS Composite Films

**Figure 15:**
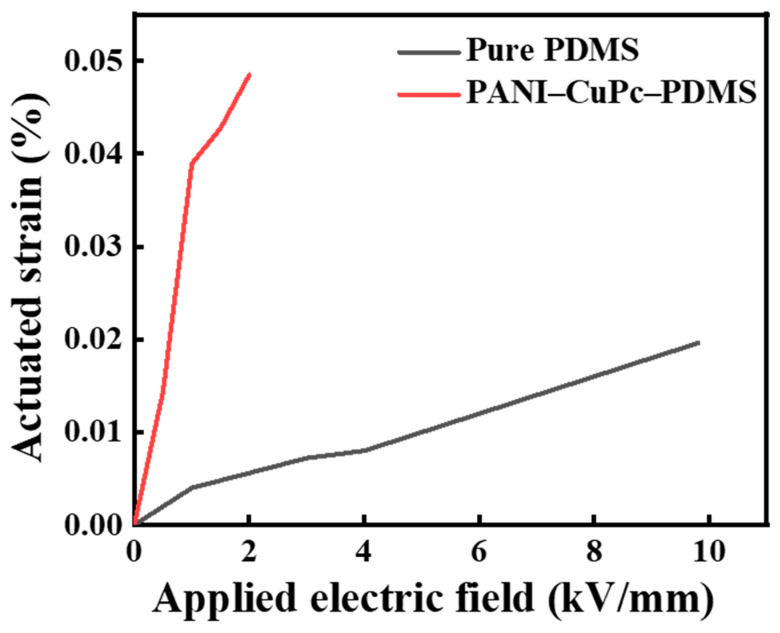
The electro-deformation of the PANI/CuPc/PDMS composite films in the field intensity range of 0 kV∙mm^−1^–10 kV∙mm^−1^.

Figure 15 shows the strain of the PANI/CuPc/PDMS composite film under different electric field intensities. It can be seen that the strain of both the composite materials and the pure PDMS increases with the increase in the electric field intensity. At the same electric field strength, the strain of the PANI/CuPc/PDMS composite is slightly higher than that of the pure PDMS, and the strain at low electric field strength (2 kV/mm) can be increased by nearly five times.

## 4. Conclusions

In this work, a high dielectric all-organic three-phase composite film was prepared by fabricating micro-network structures consisting of PANI and CuPc particles in a PDMS matrix under an applied electric field. The effects of electric field parameters on the formation of the self-assembled structures were studied. Under the electric field intensity of 2000 V/mm and the frequency of 100 Hz, the network structure formed by the CuPc particles and PANI particles is the densest and perfect. The dielectric constant of the three-phase composite film prepared after electric field orientation is higher than that of the two-phase composite film. The synergistic effect of the PANI and CuPc particles can improve the dielectric constant of the composite film. The dielectric constant of the assembled PANI/CuPc/PDMS three-phase composite film can reach 140 at 100 Hz when the content of CuPc is 4 wt% and the content of PANI is 2.5 wt%. The modulus of the three-phase composite film of PANI/CuPc/PDMS is kept below 2 MPa, which makes this composite a good candidate for electro-deformation materials. The strain at low electric field strength (2 kV/mm) of the self-assembled PANI/CuPc/PDMS three-phase composite films increased by nearly five times. By utilizing electric field-assisted self-assembly technology, the ordered arrangement of the PANI and CuPc particles in PDMS was achieved, which improved the dielectric properties of the composite material at a lower filler content. This provides new material selection and preparation strategies for the application of DEs in the fields of flexible electronics and soft robots. The future research direction will optimize the phase boundary problem between the particles and the matrix, combining theoretical simulation and computer simulation to comprehensively explain the influence of particle arrangement on material properties.

## Data Availability

The raw data supporting the conclusions of this article will be made available by the authors upon request.
